# Moxifloxacin modulates inflammation during murine pneumonia

**DOI:** 10.1186/1465-9921-15-82

**Published:** 2014-07-17

**Authors:** Christoph Beisswenger, Anja Honecker, Andreas Kamyschnikow, Markus Bischoff, Thomas Tschernig, Robert Bals

**Affiliations:** 1 Department of Internal Medicine V – Pulmonology, Allergology and Respiratory Critical Care Medicine, Saarland University, Homburg, Germany; 2 Institute of Medical Microbiology and Hygiene, Saarland University Hospital, Homburg, Germany; 3 Institute of Anatomy and Cell Biology, Saarland University Hospital, Homburg, Germany

**Keywords:** Moxifloxacin, Pneumonia, Infection, Inflammation

## Abstract

**Background:**

Moxifloxacin is a synthetic antibacterial agent belonging to the fluoroquinolone family. The antimicrobial activity of quinolones against Gram-positive and Gram-negative bacteria is based on their ability to inhibit topoisomerases. Quinolones are described to have immunomodulatory features in addition to their antimicrobial activities. It was the goal of this study to examine whether a short term treatment with moxifloxacin modulates the inflammation during a subsequently induced bacterial infection in an animal model.

**Methods:**

Mice were treated with moxifloxacin or saline for two consecutive days and were subsequently intranasally infected with viable or heat-inactivated bacterial pathogens (*Streptococcus pneumoniae*, *Pseudomonas aeruginosa*) for 6 and 24 hours. Measurements of cytokines in the lungs and plasma were performed. Alveolar cells were determined in bronchoalveolar lavage fluits.

**Results:**

The inflammation was increased after the inoculation of viable bacteria compared to inactivated bacteria. Numbers of total immune cells and neutrophils and concentrations of inflammatory mediators (e.g. KC, IL-1β, IL-17A) were significantly reduced in lungs of moxifloxacin-treated mice infected with inactivated and viable bacterial pathogens as compared to infected control mice. Plasma concentrations of inflammatory mediators were significantly reduced in moxifloxacin-treated mice. Immunohistochemistry showed a stronger infiltrate of TNF-α-expressing cells into lungs of saline-treated mice infected with viable *P. aeruginosa* as compared to moxifloxacin-treated mice.

**Conclusions:**

These data show that in this pneumonia model moxifloxacin has anti-inflammatory properties beyond its antibacterial activity.

## Introduction

Respiratory tract diseases, such as pneumonia, cystic fibrosis (CF), and chronic obstructive pulmonary disease (COPD), are worldwide a leading cause of mortality [1]. Acute and chronic inflammations leading to destruction of lung tissue are characteristic hallmarks of these diseases. For example, in severe pneumonia the infecting pathogens and the inflammatory response of the host cause a breach of epithelial and endothelial barriers [2,3]. As a pathophysiological consequence, bacteria, components of bacteria (e.g. LPS, lipoproteins), and inflammatory mediators translocate from epithelial surfaces into subepithelial compartments and into the bloodstream. In subepithelial compartments, bacteria and components of bacteria are recognized by immune cells. Activated immune cells further contribute to excessive inflammation by releasing inflammatory mediators and directing additional effector cells from the bloodstream into the parenchyma of the lung [4]. Thus, formation of edema and impaired pulmonary gas exchange are a hallmark of severe pneumonia [2,3]. Acute and chronic inflammation and infection of the lung also contribute to tissue destruction and loss of lung function in COPD and CF [1,5]. Bacterial and viral infections are associated with exacerbations of COPD [1,6]. In CF, ongoing inflammation and activation of immune mechanisms is evoked by colonizing bacteria, such as *P. aeruginosa*[7].

Moxifloxacin is a synthetic antibacterial agent belonging to the fluoroquinolone family. Moxifloxacin is well established in the treatment of patients suffering from pneumonia [8] or acute exacerbation of COPD [9]. The minimum inhibitory concentrations (MICs) for moxifloxacin have been shown to be between 0.8 to 2 mg/l for *P. aeruginosa* (e.g. strain PAO1) and 0.06 to 0.5 mg/l for *S.* pneumoniae [10-13]. The antimicrobial activities of fluoroquinolones such as moxifloxacin are based on their ability to inhibit topoisomerases (e.g. DNA gyrase and topoisomerase IV). There is strong evidence that fluoroquinolones additionally exhibit immunomodulatory functions during inflammation and microbial infection besides their bactericidal activities [8]. In vitro studies showed that clinically relevant concentrations of moxifloxacin inhibit the synthesis of inflammatory mediators (e.g. IL-1, TNF-α, IL-6, IL-8) in human peripheral blood mononuclear cells and in the monocytic cell line THP-1 stimulated with LPS, LTA, heat-inactivated bacteria, and *Aspergillus fumigatus*. This is likely due to the inhibition of NF-κB- and MAP kinase-dependent signaling pathways [14-16]. In vitro studies further revealed that moxifloxacin inhibits the activation of MAP kinase and NF-kB signaling cascades, the synthesis of nitric oxide, and the expression of the chemokines IL-6 and IL-8 in human respiratory epithelial cell lines stimulated with inflammatory cytokines [17,18]. To date, it is not sufficiently understood how fluoroquinolones modulate inflammation and particularly the activation of cellular signaling molecules such as NF-κB- or MAP kinases. However, it has been shown that fluoroquinolones affect phosphodiesterases and intracellular cAMP levels [19,20]. In addition, it has also been suggested that the immunomodulatory effects of fluoroquinolones are due to the inhibition of topoisomerases, which leads to a stress response in eukaryotic cells [8,19,21].

It was the goal of this study to gain insight into the immunomodulatory features of moxifloxacin during murine pneumonia. To study whether moxifloxacin modulates the inflammatory response of the host mice were treated with moxifloxacin before infection of the lung with viable or heat-inactivated bacterial pathogens. Infection with heat-inactivated bacteria allowed examining the effects of moxifloxacin treatment on the inflammatory response of the host without being affected by the impact of moxifloxacin on the viability of the bacteria. To demonstrate whether the immunomodulatory features of moxifloxacin depend on the infecting bacterial species we chose to infect mice with distantly related Gram-negative and Gram-positive bacterial species (*S. pneumoniae*, *P. aeruginosa*) which are known to have different susceptibility to moxifloxacin. The results further demonstrate whether a bacterial stimulation using inactivated bacteria differs in comparison to an infection caused by viable bacteria.

## Methods

### Mouse experiments

*P. aeruginosa* strain PAO1 was grown overnight at 37°C on LB agar plates. Bacterial cells were taken from the plate, resuspended in LB medium, and incubated for 2-4 hours at 37°C and 150 rpm. A type 6A clinical isolate of *S. pneumoniae* was cultured as described before [22]. After washing with PBS, bacteria were adjusted to OD_600_ = 1 and heat-inactivated for 10 min at 95°C or used directly for infection. Mice (C57BL/6N) were maintained under a pathogen-free condition. All animal experiments were approved by the Landesamt für Soziales, Gesundheit und Verbraucherschutz of the State of Saarland following the national guidelines for animal treatment. To examine the effect of moxifloxacin on the inflammatory response of the host during bacterial pneumonia mice were injected i.p. with 100 mg/kg moxifloxacin twice a day for two consecutive days. Control mice received saline. Two hours after the final moxifloxacin injection, moxifloxacin-treated and control animals were infected with bacteria [23]. Mice were slightly anesthetized by i.p. injection of 2.6 mg of ketaminhydrochloride (Ketanest; Pfizer, Germany) and 0.18 mg of xylazinhydrochloride (Rompun; Bayer, Germany) per mouse and infected intranasally with heat-inactivated (low dose: 1 × 10^6^ to 5 × 10^6^ CFU, high dose 1 × 10^7^ to 5 × 10^7^ CFU) and viable (1 × 10^7^ to 5 × 10^7^) *P. aeruginosa* and with heat-inactivated and viable *S. pneumoniae* (5 × 10^6^ to 1 × 10^7^) or with PBS alone as control. At least five mice per group were infected with viable or inactivated bacteria or treated with PBS as control. Six or 24 hours after the bacterial infection, mice were euthanized, the tracheae were cannulated and a bronchoalveolar lavage (BAL) was performed with 1 ml of PBS flushed three times into the lungs. Lungs were removed for immunohistochemistry. BAL fluids were centrifuged at 4°C to obtain BAL cells and cell-free supernatants. Alveolar cells were suspended in 1 ml of PBS. Total cell numbers were determined and cytospins were prepared. Macrophages, neutrophils, lymphocytes, and eosinophils were differentiated by light microscopy. Blood was collected by cardiac puncture into EDTA (15 μl, 0.8 M) containing 1 ml syringes and plasma was removed after centrifugation. Plasma and BAL fluids were kept at -80°C until use.

### Determination of cytokine concentrations

Concentrations of the inflammatory cytokines KC and IL-1β were assessed by enzyme-linked immunosorbent assay (ELISA) using a Tecan Ultra 384 ELISA reader and the software Magellan (Tecan, Germany). All ELISA kits were purchased from R&D Systems (UK) and used as instructed by the manufacturer. IL-17A and IL-10 were assessed by cytometric bead array (CBA, BD Bioscience, Germany).

### RNA isolation and realtime RT-PCR

Total RNA from lungs was isolated using TRIzol reagents (Life Technologies, Germany) according to the manufacturer's manual. 1 μg of total RNA was reversely transcribed to cDNA using the RevertAid First Strand cDNA Synthesis Kit (Thermo Scientific, Germany) according to the manufacturer's manual. qRT-PCR was performed with a reaction mix including the SensiMix SYBR & Fluorescein Kit (Bioline, Germany), using the iCycler (Bio-Rad Laboratories, Germany) with a 2-step protocol (15 sec at 95°C/45 sec at 60°C). Specificity of amplification was controlled by melt curve analysis and gelelectrophoresis. RT-PCR results were analysed with the ΔΔCT method [24], using GAPDH as an internal standard to normalize mRNA amounts.

### Immunohistochemistry

Lungs of mice were embedded in paraffin and immunohistochemistry was performed as described earlier [25]. TNF-α was detected with primary antibodies (Abcam, UK) diluted 1:100 in TBS (Tris-buffered saline). A biotinylated anti-rabbit secondary antibody was added (DAKO, Denmark), followed by avidin-horseradish peroxidase reagent (EnVision System (AEC), DAKO, Denmark). Imaging was performed using the software Cell Sense Dimension (Olympus, Germany). TNF-α staining was quantified (% area) using ImageJ software.

### Statistical analysis

Values are displayed as mean ± SEM. Comparisons between groups were analyzed by ANOVA (Newman-Keuls multiple comparison test). Results were considered statistically significant for p < 0.05. All statistical tests were performed using the software Prism (GraphPad Software, San Diego, CA, USA).

## Results

### Moxifloxacin treatment affects the influx of immune cells into lungs during bacterial pneumonia

Moxifloxacin treatment resulted in an increased clearance of *P. aeruginosa* and *S. pneumoniae* in the lungs of mice infected with viable bacteria indicating a bactericidal activity of moxifloxacin in our pneumonia model against both bacterial species. No viable bacteria were detected in BAL fluids of moxifloxacin-treated mice infected with 5 × 10^6^ CFU of *S. pneumoniae* 24 hours post infection, whereas 7 × 10^3^ (±3 × 10^3^) CFU of viable *S. pneumoniae* were determined in BAL fluids of saline-treated control mice. In mice infected with 10^7^ CFU of *P. aeruginosa* for 6 hours, 4 × 10^5^ (±2 × 10^5^) CFU were detected in BAL fluids of saline-treated control mice and 9 × 10^2^ (±1.3 × 10^3^) CFU in BAL fluids of moxifloxacin-treated mice.

**Figure 1:**
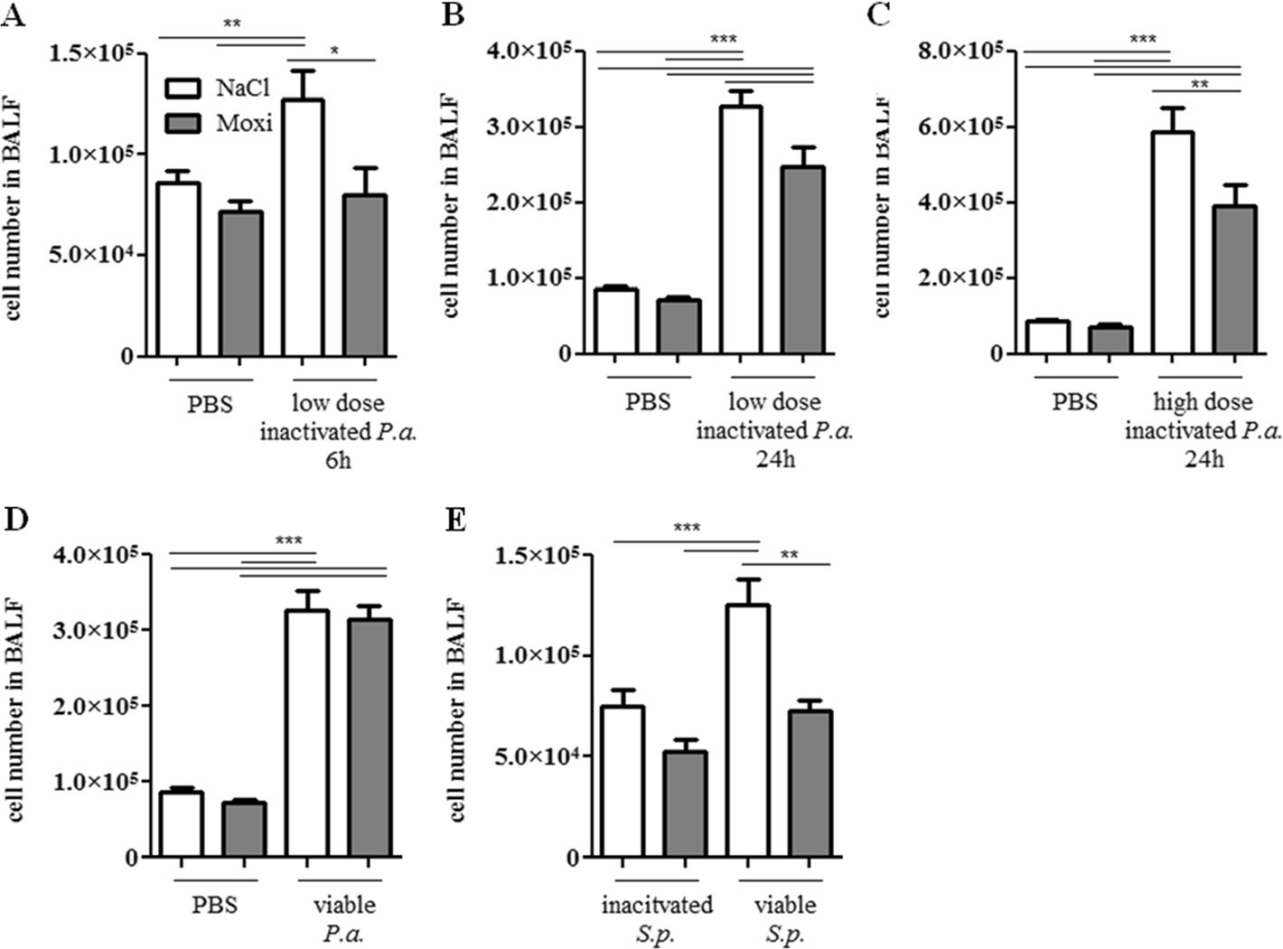
**Moxifloxacin treatment results in a reduced influx of immune cells into the lung during bacterial pneumonia.** BAL fluids of moxifloxacin- and saline-treated mice were collected 6 or 24 h post intranasal infection with heat-inactivated *P. aeruginosa***(A/B/C)**, viable *P. aeruginosa***(D)**, and heat-inactivated or viable *S. pneumoniae***(E)**. Total cell numbers were determined. Data are shown as mean ± SEM. Bars indicate significant differences of *p < 0.05, **p < 0.01, and ***p < 0.001, (n ≥ 5 for each group).

**Figure 2:**
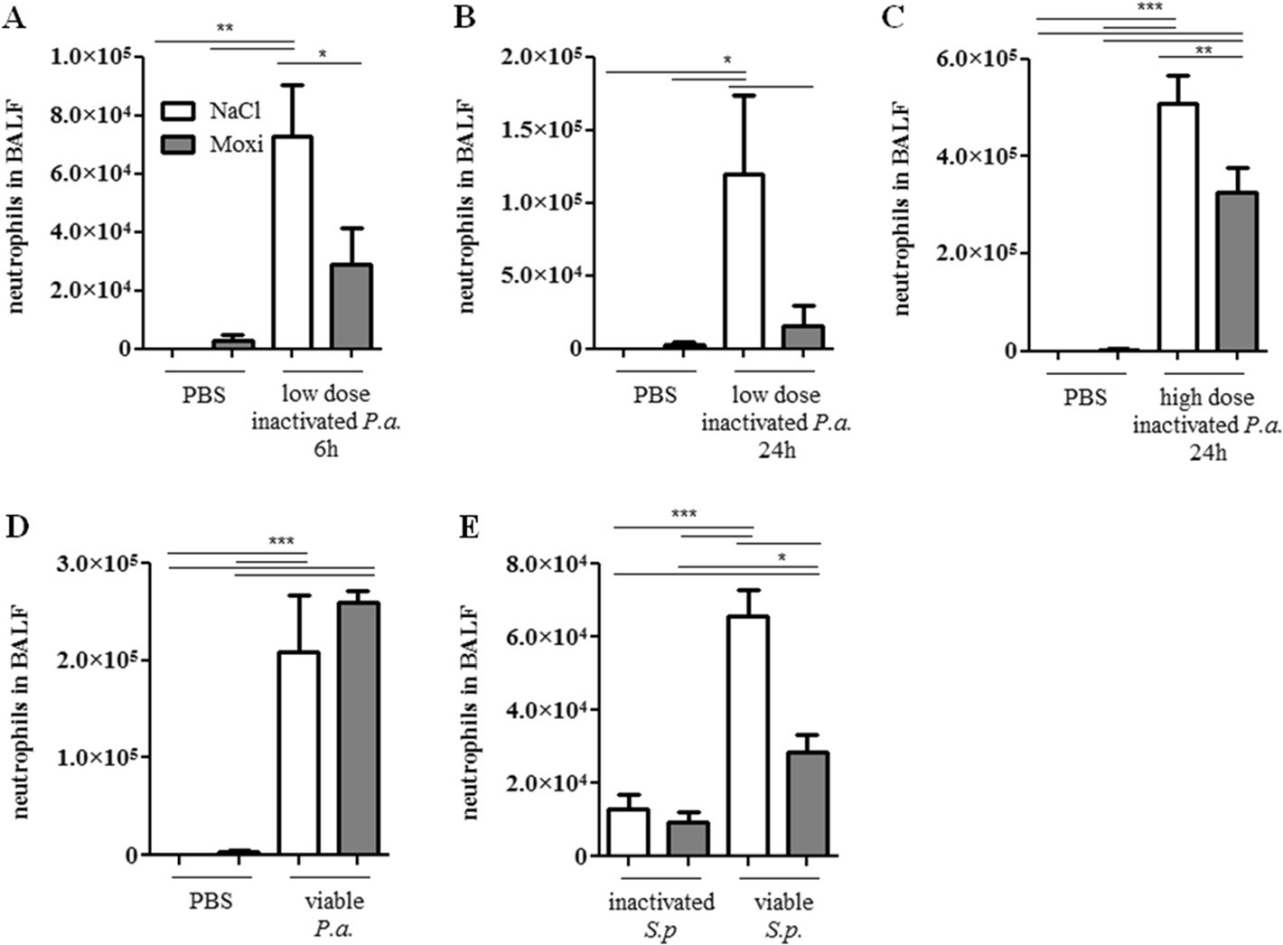
**Moxifloxacin treatment results in a reduced influx of neutrophils into the lung during bacterial pneumonia.** BAL fluids of moxifloxacin- and saline-treated mice were collected 6 or 24 h post intranasal infection with heat-inactivated *P. aeruginosa***(A/B/C)**, viable *P. aeruginosa***(D)**, and heat-inactivated or viable *S. pneumoniae***(E)**. Total numbers of neutrophils were determined. Data are shown as mean ± SEM. Bars indicate significant differences of *p < 0.05, **p < 0.01, and ***p < 0.001, (n ≥ 5 for each group).

Moxifloxacin treatment did not affect the levels of total immune cells (Figure 1) and neutrophils (Figure 2) in the lungs of mice in the absence of bacterial infection. Infection with heat-inactivated and viable bacteria resulted in a neutrophil-dominated inflammatory response in the lung. The infection with heat-inactivated and viable *P. aeruginosa* and *S. pneumoniae* led to enhanced numbers of total immune cells (Figure 1) and neutrophils (Figure 2) in BAL fluids 6 and 24 hours post infection as compared to non-infected control groups. Furthermore, in the case of *S. pneumoniae*, viable bacteria induced a significantly enhanced influx of total immune cells (Figure 1E) and neutrophils (Figure 2E) into lungs as compared to heat-inactivated *S. pneumoniae*. Interestingly, treatment with moxifloxacin affected the levels of total immune cells and neutrophils in the lungs of mice infected with viable and heat-inactivated bacteria. Treatment with moxifloxacin, as compared to saline, resulted in significantly reduced levels of total immune cells (Figure 1A to C) and neutrophils (Figure 2A to C) in BAL fluids of mice infected with a low dose or a high dose of heat-inactivated *P. aeruginosa* or with viable *S. pneumoniae* (Figures 1E and 2E). There was no difference in the numbers of total immune cells in BAL fluids between moxifloxacin- and saline-treated mice infected with viable *P. aeruginosa* (Figures 1D and 2D). Levels of macrophages and lymphocytes in BAL fluids were not affected by treatment with moxifloxacin (data not shown).

These data suggest that moxifloxacin attenuates the inflammatory response in the lung during murine pneumonia partly independent of its bactericidal activity.

### Expression of inflammatory cytokines in lungs of moxifloxacin-treated mice

**Figure 3:**
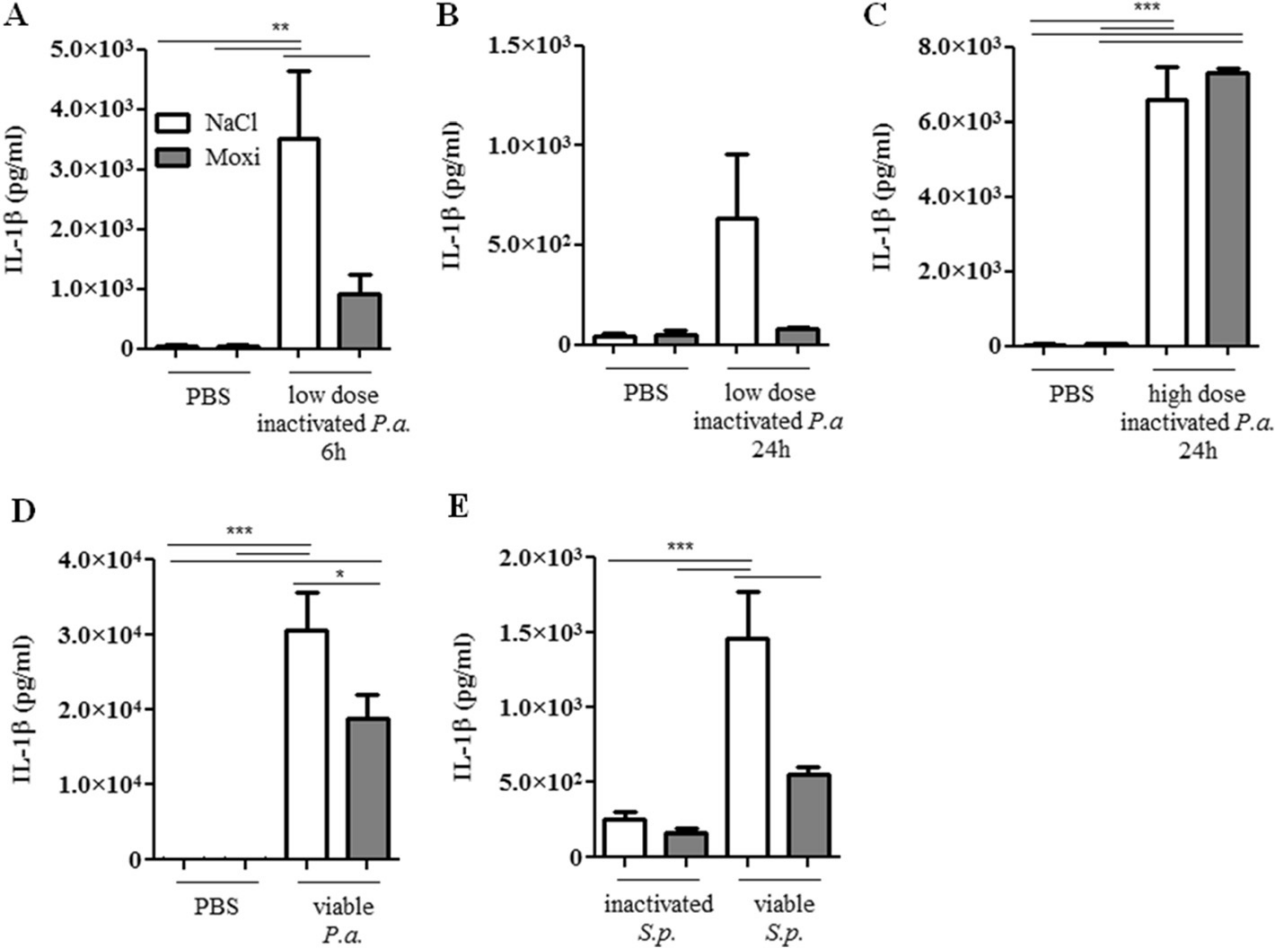
**Effect of moxifloxacin on IL-1β concentrations in lungs of infected mice.** IL-1β concentrations were measured in lungs of moxifloxacin- and saline-treated mice 6 or 24 h post intranasal infection with heat-inactivated *P. aeruginosa***(A/B/C)**, viable *P. aeruginosa***(D)**, and heat-inactivated or viable *S. pneumoniae***(E)**. Data are shown as mean ± SEM. Bars indicate significant differences of *p < 0.05, **p < 0.01, and ***p < 0.001 (n ≥ 5 for each group).

**Figure 4:**
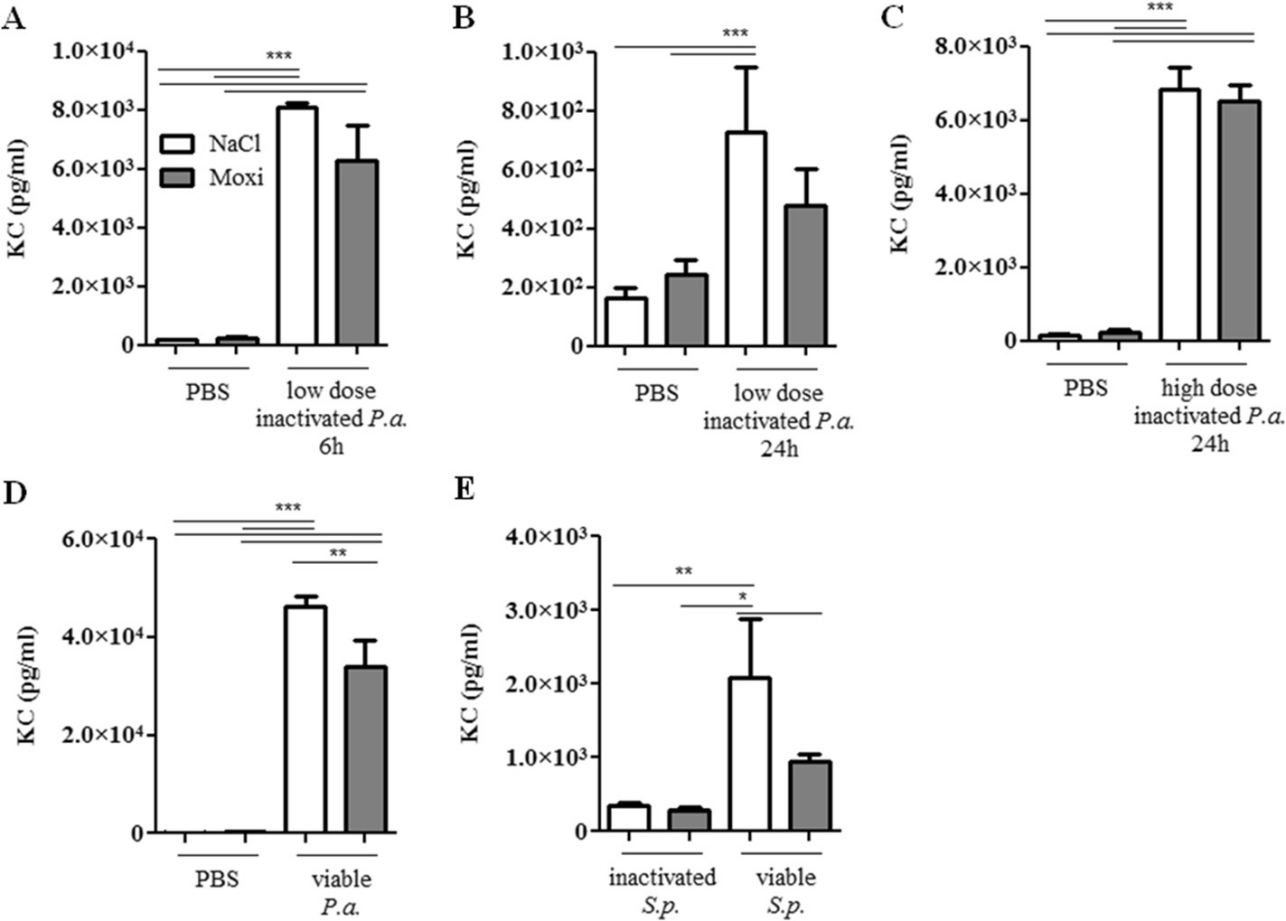
**Effect of moxifloxacin on KC concentrations in lungs of infected mice.** KC concentrations were measured in lungs of moxifloxacin- and saline-treated mice 6 or 24 h post intranasal infection with heat-inactivated *P. aeruginosa***(A/B/C)**, viable *P. aeruginosa***(D)**, and heat-inactivated or viable *S. pneumoniae***(E)**. Data are shown as mean ± SEM. Bars indicate significant differences of *p < 0.05, **p < 0.01, and ***p < 0.001 (n ≥ 5 for each group).

**Figure 5:**
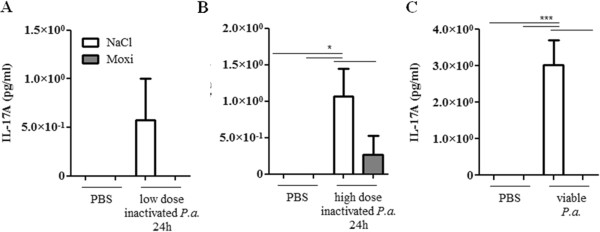
**Effect of moxifloxacin on IL-17A concentrations in lungs of infected mice.** IL-17A concentrations were measured in BAL fluids of moxifloxacin- and saline-treated mice 6 or 24 h post intranasal infection with heat-inactivated *P. aeruginosa***(A/B)** or viable *P. aeruginosa***(C)**. Data are shown as mean ± SEM. Bars indicate significant differences of *p < 0.05, **p < 0.01, and ***p < 0.001 (n ≥ 5 for each group).

**Figure 6:**
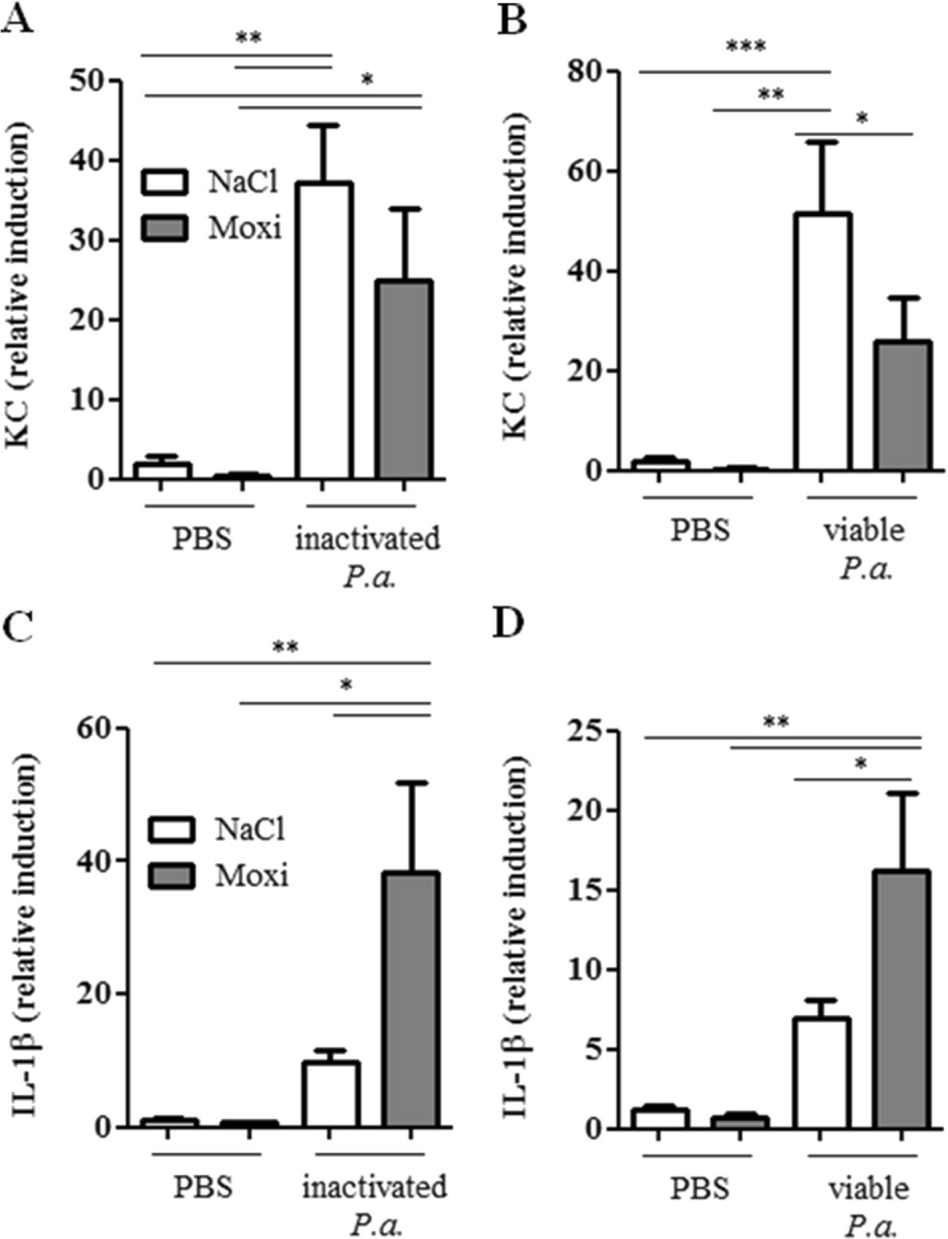
**Moxifloxacin affects the expression of inflammatory cytokines in lungs.** The relative expression of KC **(A/B)** and IL-1β **(C/D)** was measured in whole lungs of moxifloxacin- and saline-treated mice 6 post intranasal infection with heat-inactivated *P. aeruginosa***(A/C)** or viable *P. aeruginosa***(B/D)** via qRT-PCR. Data are shown as mean ± SEM. Bars indicate significant differences of *p < 0.05, **p < 0.01, and ***p < 0.001 (n ≥ 5 for each group).

**Figure 7:**
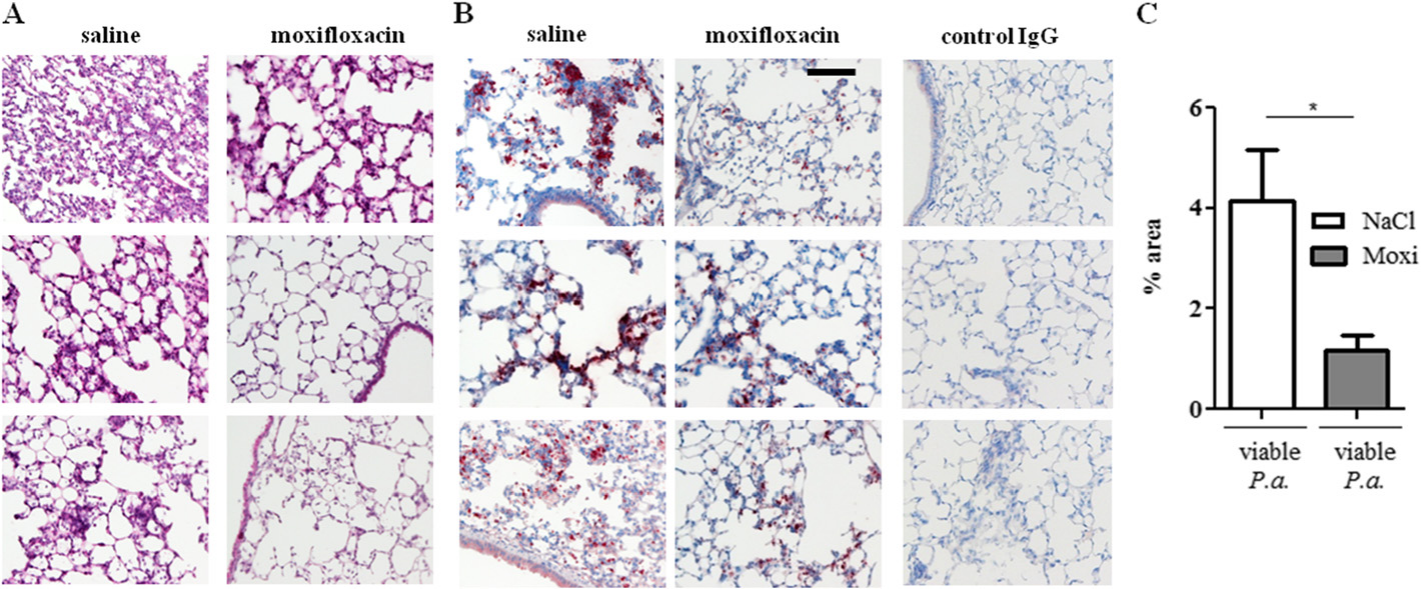
**Moxifloxacin treatment results in a reduced infiltrate of TNF-α-expressing cells.** Moxifloxacin- and saline-treated mice were infected intranasally with viable *P. aeruginosa* for 6 h. **(A)** Hematoxylin and eosin staining. **(B)** TNF-α is shown by immunohistochemistry. Scale bar represents 200 μm. Samples from three different animals per group are shown. **(C)** Quantification of TNF-α staining in 4 slides per mouse. Bar indicates significant difference of p < 0.05.

Next, we examined whether a treatment with moxifloxacin modulates the expression of inflammatory mediators in the lung upon bacterial infection. Moxifloxacin per se did not affect the levels of the inflammatory mediators IL-1β (Figure 3), KC (the functional homologue of IL-8 in mice, Figure 4) and IL-17A (Figure 5) in the lungs of mice in the absence of bacterial infection. Concentrations of IL-1β, KC and IL-17A were significantly increased in lungs of mice infected with heat-inactivated and viable *P. aeruginosa* and *S. pneumoniae* as compared to the concentrations in lungs of non-infected control mice. Furthermore, infection with viable bacteria induced significantly enhanced expression of these cytokines as compared to infection with heat-inactivated bacteria. Treatment with moxifloxacin resulted in a reduced expression of IL-1β, KC and IL-17A in lungs of mice infected with viable bacteria, whereas the levels of these cytokines in lungs of mice infected with heat-inactivated bacteria were only partially affected by moxifloxacin as compared to saline. IL-β concentrations were reduced in lung homogenates of moxifloxacin-treated mice infected for 6 hours with heat-inactivated *P. aeruginosa* (Figure 3A) and viable *P. aeruginosa* (Figure 3D) and in lung homogenates of mice infected with viable *S. pneumoniae* for 24 hours (Figure 3E). IL-1β could only be detected in BAL fluids from saline-treated mice infected with viable *P. aeruginosa* (1203 ng/ml ± 126), whereas IL-1β concentrations were below the detection limit in BAL fluids of moxifloxacin-treated mice infected with viable *P. aeruginosa* and in BAL fluids of mice infected with heat-inactivated bacteria (data not shown). Treatment with moxifloxacin resulted in significantly reduced concentrations of KC in lung homogenates of mice infected with viable *P. aeruginosa* (Figure 4D) and *S.* pneumonia (Figure 4E) as compared to saline-treated mice. KC concentrations were also significantly (p < 0.001) reduced in BAL fluids from moxifloxacin-treated mice infected with viable *P. aeruginosa* (5.4 × 10^4^ ng/ml ± 1533) as compared to infected saline-treated mice (2.1 × 10^4^ ng/ml ± 7507). IL-17A concentrations were significantly reduced in BAL fluids of moxifloxacin-treated mice infected with a high dose of heat-inactivated for 24 hours (Figure 5B) and viable *P. aer*uginosa (Figure 5C) as compared to the corresponding saline-treated mice. IL-17A was below the detection limit in mice treated with a low dose of inactivated *P. aeruginosa* for 6 hours (data not shown). Levels of the anti-inflammatory cytokine IL-10 in BAL fluids were below the detection limit (10 pg/ml) in infected and non-infected mice (data not shown). Relative mRNA expression levels of inflammatory mediators were determined in whole lungs of mice infected with inactivated and viable *P. aeruginosa* for 6 hours. In case of infection with inactivated *P. aeruginosa*, expression of KC was slightly enhanced in lungs of saline-treated mice compared to moxifloxacin-treated mice (Figure 6A). Infection with viable *P. aeruginosa* resulted in significantly increased expression levels of KC in lungs of saline-treated mice as compared to moxifloxacin-treated mice (Figure 6B). The expression of IL-β was significantly increased in moxifloxacin-treated mice infected with inactivated and viable *P. aeruginosa* (Figure 6C and D). No significantly increased levels of expression could be determined for IL-17 6 hours post infection by qRT-PCR (data not shown). In addition, infection with viable *P. aeruginosa* for 6 hours resulted in an infiltrate of TNF-α-expressing cells into lungs. Figure 7 shows representative hematoxylin/eosin and TNF-α staining of lung sections from areas with strong and moderate cell infiltrates. The infiltrate of TNF-α-expressing cells into lungs of saline-treated mice was significantly increased as compared to moxifloxacin-treated mice (Figure 7C).

### Concentrations of KC in blood of mice treated with moxifloxacin

**Figure 8:**
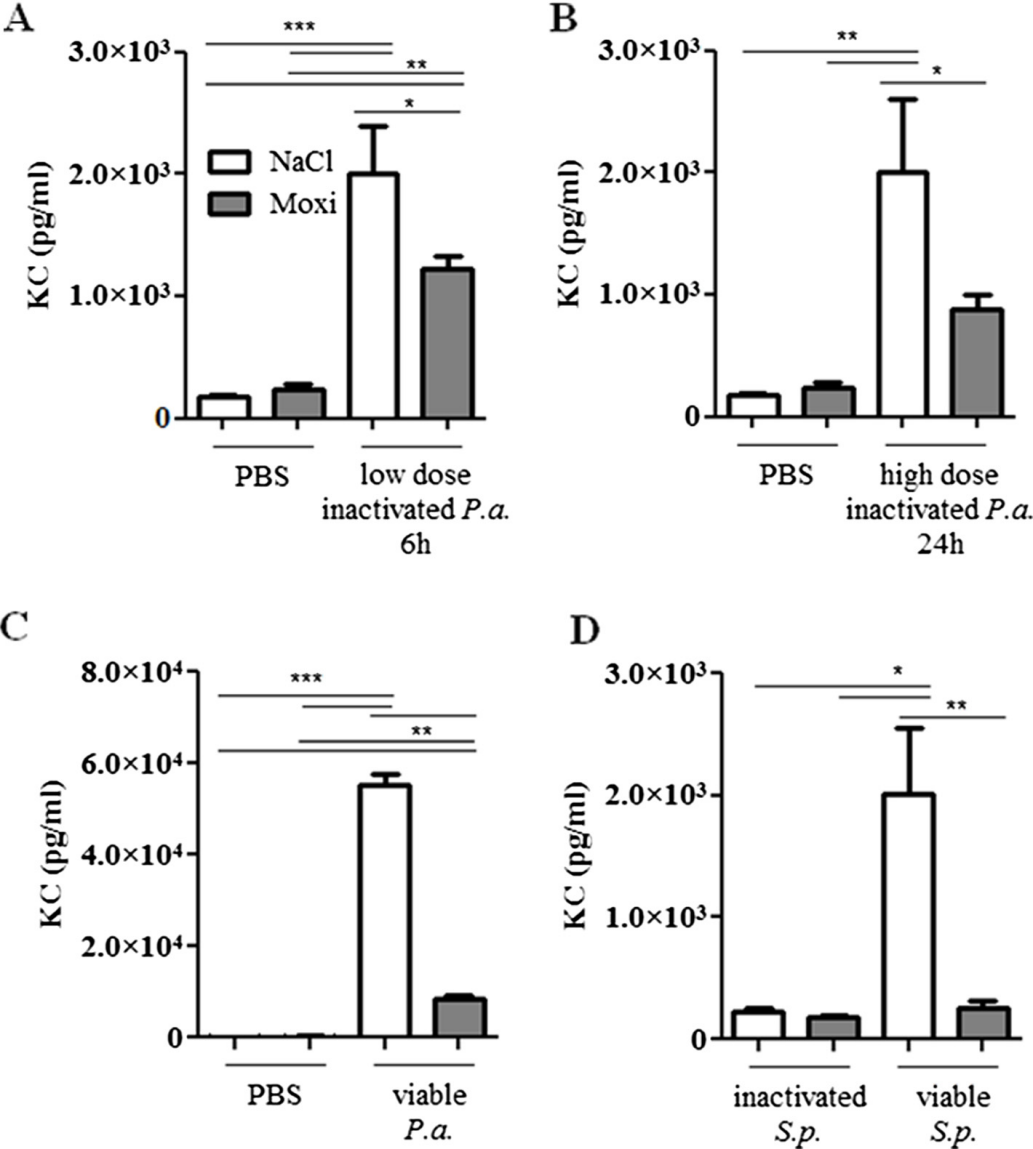
**Levels of KC are reduced in blood of mice treated with moxifloxacin.** KC concentrations were measured in plasma of moxifloxacin and saline-treated mice infected intranasally with heat-inactivated *P. aeruginosa***(A/B)**, viable *P. aeruginosa***(C)**, and heat-inactivated or viable *S. pneumoniae***(D)** for 6 or 24 h. Bars indicate significant differences of *p < 0.05, **p < 0.01, and ***p < 0.001 (n ≥ 5 for each group).

To determine whether moxifloxacin treatment affects cytokine levels in blood during bacterial pneumonia KC concentrations were measured in blood plasma. Treatment with moxifloxacin per se did not affect concentrations of KC in plasma in the absence of bacterial infection (Figure 8). KC concentrations were significantly increased in plasma of mice infected with heat-inactivated (Figure 8A and B) and viable (Figure 8C and D) bacteria as compared to non-infected control mice. Infection with viable *P. aeruginosa* and *S. pneumoniae* resulted in significantly enhanced concentrations of KC as compared to infection with heat-inactivated bacteria. Furthermore, KC concentrations were significantly reduced in plasma of moxifloxacin-treated mice infected with heat-inactivated *P.* aeruginosa (Figure 8A and B), live *P. aeruginosa* (Figure 8C) and live *S. pneumoniae* (Figure 8D) as compared to the concentrations in the plasma of the corresponding saline-treated control mice. KC concentrations in plasma of mice infected with a low dose of heat-inactivated *P. aeruginosa* for 24 hours were not significantly increased in saline or moxifloxacin-treated mice (data not shown). Thus, treatment with moxifloxacin results in reduced levels of inflammatory cytokines in the blood stream during murine pneumonia.

## Discussion

This study focused on the immunomodulatory functions of the fluoroquinolone moxifloxacin. The principle finding is that moxifloxacin attenuates local and systemic inflammation during bacterial pneumonia in mice and that the anti-inflammatory properties of moxifloxacin depend on both its bactericidal and non-bactericidal activities.

Moxifloxacin is well established in the clinical treatment of patients suffering from community-acquired pneumonia [26] and acute exacerbation of COPD [21]. In addition, an immunomodulatory effect of moxifloxacin is assumed in clinical situations [8]. Kazama and colleagues, for instance, reported an interesting case of a woman with a dual lung infection caused by *Mycoplasma pneumoniae* and *Bordetella pertussis*. The clinical course was unaffected during a therapy with three different antibiotics in series. Only the subsequent treatment with moxifloxacin led to a dramatic clinical improvement and the disappearance of the migratory pulmonary infiltrates. The authors postulated a immunomodulatory role of moxifloxacin suppressing the lymphocyte activity [27]. Furthermore, several in vitro studies showed that moxifloxacin attenuates the inflammatory response in diverse cell types (e.g. monocytes, respiratory epithelial cells) induced by microbial stimuli and inflammatory mediators by inhibiting the activation of MAP kinases and NF-kB [14-18,28]. Moxifloxacin reduced the cytokine-induced activation of the transcription factor NF-κB and MAP kinases and the synthesis of nitric oxide in the alveolar epithelial cell line A549 [17] and the TNF-α-induced NF-κB- and MAP-kinase-dependent expression of inflammatory mediators (IL-6, IL-8) in cystic fibrosis epithelial cells [18]. Moxifloxacin also inhibited the activation of MAP kinases and NF-κB and the release of inflammatory mediators in human monocytes in response to bacterial stimuli [14-16].

To study whether moxifloxacin reduces the inflammatory response of the host to microbial pathogens as seen in the in vitro studies mentioned above we treated mice with moxifloxacin prior to infection with viable or heat-inactivated bacterial pathogens. In line with the in vitro studies, our murine pneumonia model showed that systemic treatment with moxifloxacin results in an attenuated inflammatory response with reduced levels of pro-inflammatory mediators and neutrophils in the lungs of mice infected with viable and heat-inactivated *S. pneumoniae* and *P. aeruginosa*. Even though the inflammation was more accentuated after the inoculation of viable bacteria than of inactivated bacteria and moxifloxacin was more efficient in suppressing the strong inflammation induced by viable bacteria, infection with inactivated bacteria demonstrated that the anti-inflammatory properties of moxifloxacin partly depend on its non-bactericidal activities. A protective function of moxifloxacin apart from its bactericidal activity could also be determined in a murine pneumonia model with *Candida albicans* in which treatment with moxifloxacin resulted in reduced levels of inflammatory mediators in the lung during the course of infection [28]. In that report, the beneficial reduction of inflammation in the *Candida-albicans*-induced pneumonia model could not be explained by an antifungal effect of moxifloxacin. This further supports an anti-inflammatory potency of moxifloxacin completely separate from its antimicrobial effect. As moxifloxacin modulated the inflammatory response to distantly related Gram-negative and Gram-positive bacteria with different susceptibility to moxifloxacin and to fungal species, treatment with moxifloxacin may be beneficial for patients with infection-related lung diseases by reducing the inflammatory burden of the lung independent of the infecting pathogen and its susceptibility to the drug.

It is difficult to deduce the specific cell types affected by moxifloxacin from this in vivo study. It seems to be reasonable that moxifloxacin directly effects the activation of immune cells (e.g. alveolar macrophages) by bacterial pathogens in the infected lungs as observed in the mentioned in vitro studies, since IL-17A is expressed by immune cells and not by epithelial cells [29] and macrophages are a main source of IL-1β during acute infection of the lung [25]. The finding that treatment with moxifloxacin resulted in reduced IL-1β protein concentrations but in increased mRNA transcription in the lungs of mice infected with viable bacteria whereas KC transcription and protein concentrations were reduced in lungs of moxifloxacin-treated mice suggests that moxifloxacin modulates inflammation at different levels. Additional studies are needed to examine whether moxifloxacin-treatment modulates activation of the inflammasome that mediates the activation and release of IL-1β and whether moxifloxacin-treatment differentially affects signaling pathways required for mRNA-transcription and stabilization of specific cytokins. Furthermore, the activation of respiratory epithelial cells also may be modulated by moxifloxacin as epithelial cells are a source of KC during bacterial pneumonia [25].

Moreover, viability of the bacterial pathogens per se also affected the inflammatory response of the host. Infection with viable bacteria resulted in absolute levels of pro-inflammatory mediators in the lungs and in plasma of mice that were approximately one magnitude higher as compared to infection with heat-inactivated bacteria, even though infection with heat-inactivated *P. aeruginosa* resulted in a robust influx of total immune cells and neutrophils into the lungs. In addition, moxifloxacin impacted viability of *P. aeruginosa* and *S. pneumoniae*. Fewer bacteria could be cultured from lungs of mice treated with moxifloxacin and infected with viable bacteria as compared to lungs of mice treated with saline. The bactericidal activity of moxifloxacin correlated with the inflammatory response of the host since treatment with moxifloxacin, as compared to saline, resulted in reduced levels of inflammatory mediators in lungs and in plasma of mice infected with viable *P. aeruginosa* and *S. pneumoniae* and in a reduced influx of immune cells into lungs of mice infected with *S. pneumoniae*. These results indicate that the viability of bacteria determines the inflammatory response of the host and are in line with a study of Sander et al. [30] showing that the immune system can distinguish viable from dead microorganisms by sensing prokaryotic viability-associated patterns resulting in differential release of inflammatory mediators via activation of the inflammasome, such as IL-1β. In line with this study, we found that viability of bacteria does not affect the expression of IL-1β at a transcriptional level. However, inactivation of bacteria resulted in significantly reduced protein levels of IL-β in lungs of infected mice. Thus, moxifloxacin seems to modulate inflammation during bacterial pneumonia by reducing the amount of viability-associated patterns and via mechanisms independent of its bactericidal activity.

In conclusion, we show that moxifloxacin reduces the “hyper-inflammation” during murine pneumonia. Pretreatment with moxifloxacin attenuates local and systemic inflammation during bacterial pneumonia in mice. The non-bactericidal properties may be beneficial for patients with infection-related lung diseases.

## Competing interest

The authors declare that they have no competing interest.

## Authors’ contributions

CB: designed the study, collected data, analyzed data, and wrote the manuscript. AH: collected data. AK: collected data. MB and TT: analyzed data and wrote the manuscript. RB: designed the study, analyzed data, and wrote the manuscript. All authors read and approved the final manuscript.

## References

[B1] Sethi S Infection as a comorbidity of COPD Eur Respir J 2010 35 1209 1215 10.1183/09031936.00081409 20513910

[B2] Witzenrath MB Gutbier AC Hocke B Schmeck S Hippenstiel K Berger TJ Mitchell JR DeLos Toyos S Rosseau N Suttorp H Schutte Role of pneumolysin for the development of acute lung injury in pneumococcal pneumonia Crit Care Med 2006 34 1947 1954 10.1097/01.CCM.0000220496.48295.A9 16715037

[B3] Matthay MA Ware LB Zimmerman GA The acute respiratory distress syndrome J Clin Invest 2012 122 2731 2740 10.1172/JCI60331 22850883 PMC3408735

[B4] Shaykhiev R Bals R Interactions between epithelial cells and leukocytes in immunity and tissue homeostasis J Leukoc Biol 2007 82 1 15 10.1189/jlb.0207096 17452476

[B5] Bals R Weiner DJ Wilson JM The innate immune system in cystic fibrosis lung disease J Clin Invest 1999 103 303 307 10.1172/JCI6277 9927489 PMC407907

[B6] Sethi S Murphy TF Infection in the pathogenesis and course of chronic obstructive pulmonary disease N Engl J Med 2008 359 2355 2365 10.1056/NEJMra0800353 19038881

[B7] Dubin PJ Martz A Eisenstatt JR Fox MD Logar A Kolls JK Interleukin-23-mediated inflammation in Pseudomonas aeruginosa pulmonary infection Infect Immun 2012 80 398 409 10.1128/IAI.05821-11 22025517 PMC3255685

[B8] Dalhoff A Immunomodulatory activities of fluoroquinolones Infection 2005 33 Suppl 2 55 70 16518713 10.1007/s15010-005-8209-8

[B9] Wilson R Anzueto A Miravitlles M Arvis P Alder J Haverstock D Trajanovic M Sethi S Moxifloxacin versus amoxicillin/clavulanic acid in outpatient acute exacerbations of COPD: MAESTRAL results Eur Respir J 2012 40 17 27 10.1183/09031936.00090311 22135277 PMC3393767

[B10] Lister PD Sanders CC Pharmacodynamics of moxifloxacin, levofloxacin and sparfloxacin against Streptococcus pneumoniae J Antimicrob Chemother 2001 47 811 818 10.1093/jac/47.6.811 11389113

[B11] Woodcock JM Andrews JM Boswell FJ Brenwald NP Wise R In vitro activity of BAY 12-8039, a new fluoroquinolone Antimicrob Agents Chemother 1997 41 101 106 8980763 10.1128/aac.41.1.101 PMC163668

[B12] Zhang L Li XZ Poole K Fluoroquinolone susceptibilities of efflux-mediated multidrug-resistant Pseudomonas aeruginosa, Stenotrophomonas maltophilia and Burkholderia cepacia J Antimicrob Chemother 2001 48 549 552 10.1093/jac/48.4.549 11581236

[B13] Buyck JM Tulkens PM Van BF Pharmacodynamic evaluation of the intracellular activity of antibiotics towards Pseudomonas aeruginosa PAO1 in a model of THP-1 human monocytes Antimicrob Agents Chemother 2013 57 2310 2318 10.1128/AAC.02609-12 23478951 PMC3632903

[B14] Araujo FG Slifer TL Remington JS Effect of moxifloxacin on secretion of cytokines by human monocytes stimulated with lipopolysaccharide Clin Microbiol Infect 2002 8 26 30 10.1046/j.1469-0691.2002.00374.x 11906497

[B15] Weiss T Shalit I Blau H Werber S Halperin D Levitov A Fabian I Anti-inflammatory effects of moxifloxacin on activated human monocytic cells: inhibition of NF-kappaB and mitogen-activated protein kinase activation and of synthesis of proinflammatory cytokines Antimicrob Agents Chemother 2004 48 1974 1982 10.1128/AAC.48.6.1974-1982.2004 15155187 PMC415605

[B16] Choi JH Song MJ Kim SH Choi SM Lee DG Yoo JH Shin WS Effect of moxifloxacin on production of proinflammatory cytokines from human peripheral blood mononuclear cells Antimicrob Agents Chemother 2003 47 3704 3707 10.1128/AAC.47.12.3704-3707.2003 14638469 PMC296188

[B17] Werber S Shalit I Fabian I Steuer G Weiss T Blau H Moxifloxacin inhibits cytokine-induced MAP kinase and NF-kappaB activation as well as nitric oxide synthesis in a human respiratory epithelial cell line J Antimicrob Chemother 2005 55 293 300 10.1093/jac/dkh525 15659543

[B18] Blau H Klein K Shalit I Halperin D Fabian I Moxifloxacin but not ciprofloxacin or azithromycin selectively inhibits IL-8, IL-6, ERK1/2, JNK, and NF-kappaB activation in a cystic fibrosis epithelial cell line Am J Physiol Lung Cell Mol Physiol 2007 292 L343 L352 17012372 10.1152/ajplung.00030.2006

[B19] Dalhoff A Shalit I Immunomodulatory effects of quinolones Lancet Infect Dis 2003 3 359 371 10.1016/S1473-3099(03)00658-3 12781508

[B20] Bailly S Fay M Roche Y Gougerot-Pocidalo MA Effects of quinolones on tumor necrosis factor production by human monocytes Int J Immunopharmacol 1990 12 31 36 10.1016/0192-0561(90)90065-U 1689279

[B21] Riesbeck K Forsgren A Henriksson A Bredberg A Ciprofloxacin induces an immunomodulatory stress response in human T lymphocytes Antimicrob Agents Chemother 1998 42 1923 1930 9687385 10.1128/aac.42.8.1923 PMC105711

[B22] Ratner AJ Lysenko ES Paul MN Weiser JN Synergistic proinflammatory responses induced by polymicrobial colonization of epithelial surfaces Proc Natl Acad Sci U S A 2005 102 3429 3434 10.1073/pnas.0500599102 15728393 PMC552945

[B23] Beisswenger C Kandler K Hess C Garn H Felgentreff K Wegmann M Renz H Vogelmeier C Bals R Allergic airway inflammation inhibits pulmonary antibacterial host defense J Immunol 2006 177 1833 1837 10.4049/jimmunol.177.3.1833 16849494

[B24] Pfaffl MW A new mathematical model for relative quantification in real-time RT-PCR Nucleic Acids Res 2001 29 e45 10.1093/nar/29.9.e45 11328886 PMC55695

[B25] Hess C Herr C Beisswenger C Zakharkina T Schmid RM Bals R Myeloid RelA regulates pulmonary host defense networks Eur Respir J 2010 35 343 352 10.1183/09031936.00196408 19679599

[B26] Ewig S Hecker H Suttorp N Marre R Welte T Moxifloxacin monotherapy versus ss-lactam mono- or combination therapy in hospitalized patients with community-acquired pneumonia J Infect 2011 62 218 225 10.1016/j.jinf.2011.01.009 21276814

[B27] Kazama I Tamada T Nakajima T Resolution of migratory pulmonary infiltrates by moxifloxacin in a patient with dual infection of Mycoplasma pneumoniae and Bordetella pertussis Infez Med 2012 20 288 292 23299070

[B28] Shalit I Horev-Azaria L Fabian I Blau H Kariv N Shechtman I Alteraz H Kletter Y Immunomodulatory and protective effects of moxifloxacin against Candida albicans-induced bronchopneumonia in mice injected with cyclophosphamide Antimicrob Agents Chemother 2002 46 2442 2449 10.1128/AAC.46.8.2442-2449.2002 12121916 PMC127325

[B29] Pfeifer P Voss M Wonnenberg B Hellberg J Seiler F Lepper PM Bischoff M Langer F Schafers HJ Menger MD Bals R Beisswenger C IL-17C is a mediator of respiratory epithelial innate immune response Am J Respir Cell Mol Biol 2013 48 415 421 10.1165/rcmb.2012-0232OC 23221046

[B30] Sander LE Davis MJ Boekschoten MV Amsen D Dascher CC Ryffel B Swanson JA Muller M Blander JM Detection of prokaryotic mRNA signifies microbial viability and promotes immunity Nature 2011 474 385 389 10.1038/nature10072 21602824 PMC3289942

